# Crystallographic and optical study of PbHfO_3_ crystals

**DOI:** 10.1107/S1600576717000309

**Published:** 2017-02-17

**Authors:** S. Huband, A. M. Glazer, K. Roleder, A. Majchrowski, P. A. Thomas

**Affiliations:** a Department of Physics, University of Warwick, Coventry, West Midlands CV4 7AL, UK; b Clarendon Laboratory, University of Oxford, Parks Road, Oxford OX1 3PU, UK; c Institute of Physics, University of Silesia, ulica Uniwersytecka 4, Katowice, Poland; d Institute of Applied Physics, Military University of Technology, ulica Kaliskiego 2, Warsaw, Poland

**Keywords:** birefringence imaging microscopy, lead hafnate, phase transitions, Metripol

## Abstract

The symmetry of the intermediate high-temperature phase of PbHfO_3_ has been determined unambiguously to be orthorhombic using a combination of high-resolution X-ray diffraction and birefringence imaging microscopy measurements of crystal plates. While lattice parameter measurements as a function of temperature in the intermediate phase are consistent with either orthorhombic or tetragonal symmetry, domain orientations observed in birefringence imaging microscopy measurements utilizing the Metripol system are only consistent with orthorhombic symmetry with the unit cell in the rhombic orientation of the pseudocubic unit cell.

## Abstract 2

This article details the use of birefringence imaging microscopy and high-resolution X-ray diffraction for determining the symmetries of the phases in PbHfO_3_ between room temperature and 870 K.

## 1. Introduction   

Lead hafnate is antiferroelectric at room temperature with a perovskite structure and undergoes two phase transitions as a function of temperature. The initial structural measurements on PbHfO_3_ were made using X-ray powder diffraction by Shirane & Pepinsky (1953 ▸), who suggested the room- temperature phase (*A*_1_) is orthorhombic and isostructural with the room-temperature phase of PbZrO_3_. The orthorhombic unit cell in this case is related to the pseudocubic axes^1^ by the following:
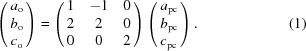
One axis (*c*_o_) is aligned along a pseudocubic perovskite axis, with the other two orthorhombic axes perpendicular but approximately 45° with respect to the other two pseudocubic axes. This type of orthorhombic cell is said to be in the rhombic orientation. The pseudocubic unit cell is then 4*a*_pc_ × 4*b*_pc_ × 2*c*_pc_ with *a*_pc_ = *b*_pc_ and the angle γ very close to 90°.

As the temperature was increased, Shirane and Pepinsky found a phase transition to an intermediate phase (*A*_2_), which occurred at 436 K, and interpreted this to be tetragonal. The phase transition to cubic symmetry was found to be at 488 K. A number of investigations have since been undertaken to determine the correct structures of the *A*_1_ and *A*_2_ phases.

Dernier & Remeika (1975 ▸) gave the *A*_1_ structure as orthorhombic with the space group likely to be *Pnam* and also suggested that *A*_2_ was rhombohedral. Two detailed structural investigations have been made, by Corker *et al.* (1998 ▸) using single-crystal X-ray and powder neutron diffraction and by Madigout *et al.* (1999 ▸) using electron diffraction and powder neutron diffraction. These are in agreement, confirming that the antiferroelectric orthorhombic *A*_1_ structure has space group *Pbam*. The intermediate phase has not been reliably determined and it has been suggested that it is orthorhombic (Leont’ev *et al.*, 1984 ▸), rhombohedral (Dernier & Remeika, 1975 ▸) or tetragonal (Shirane & Pepinsky, 1953 ▸; Fujishita & Ishikawa, 2002 ▸) or is initially orthorhombic from 433 to 453 K and tetragonal between 463 and 493 K (Kabirov *et al.*, 2011 ▸).

A range of techniques have been utilized to determine the temperature of each phase transition: these have included dielectric spectroscopy (Shirane & Pepinsky, 1953 ▸; Roleder *et al.*, 2000 ▸; Kabirov *et al.*, 2011 ▸), Raman spectroscopy (Sharma *et al.*, 1994 ▸), differential scanning calorimetry (Yoshida *et al.*, 2009 ▸), powder X-ray diffraction (Kwapulinski *et al.*, 1994 ▸) and powder neutron diffraction (Fujishita *et al.*, 2008 ▸). These measurements have suggested that the *A*_1_-to-*A*_2_ phase transition occurs between 433 and 443 K with thermal hysteresis; the phase change is between 5 and 7 K lower on cooling. The *A*_2_-to-cubic transition is between 473 and 483 K with a small thermal hysteresis of roughly 2 K.

Recently, a paper by Gorfman *et al.* (2012 ▸) detailed the use of birefringence imaging microscopy (BIM) with the Metripol system for determining the symmetry of crystals using the distribution of the measured orientations of the slow axis. When a crystal is measured in the Metripol system, with light propagating perpendicular to a pseudocubic axis, the possible orientations of the slow axis from this in-plane pseudocubic axis are as follows: 0 and 90° for tetragonal; 0, 45, 90 and 135° for orthorhombic; and 45 and 135° for rhombohedral.

In this paper we investigate the structure of each phase and the associated phase transitions using high-resolution single-crystal X-ray diffraction and BIM. The combination of these experimental techniques provides a unique insight into the structure and domain formations of the different phases of PbHfO_3_.

## 2. Experimental details   

### 2.1. Crystal growth   

A flux growth method was used for growing crystals of PbHfO_3_ to avoid stoichiometry problems introduced from its incongruent melting. A Pb_3_O_4_ self-flux was chosen instead of the commonly used PbO. Crystals grown using a PbO flux have a grey coloration, which is probably caused by an oxygen deficiency. The Pb^4+^ ions make the Pb_3_O_4_ flux slightly oxidizing, removing the grey coloration developed in PbO-based crystals. B_2_O_3_ was added to the flux to reduce the evaporation of PbO from the melt and also lower the supersaturation temperature.

Crystallization was carried out in a closed platinum crucible using a single-zone resistance furnace with low temperature gradients. The starting composition was 4.93 mol% of PbHfO_3_, 52.74 mol% of Pb_3_O_4_ and 42.33 mol% of B_2_O_3_. An initial soak at 1473 K for 24 h was followed by cooling at 3.5 K h^−1^ to 1200 K. As-grown crystals were decanted and then cooled to room temperature at a rate of 10 K h^−1^. The PbHfO_3_ crystals formed 1 × 1 × 0.1 mm plates and traces of the solidified flux were removed by etching with acetic acid (Burkovsky *et al.*, 2015 ▸).

### 2.2. Birefringence imaging microscopy   

An Oxford Cryosystems Metripol was used for measurements of the birefringence of the PbHfO_3_ single crystals. This system consists of a rotatable linear polarizer and a circular analyser as described by Glazer *et al.* (1996 ▸). The intensity, *I*, of light propagating through a crystal placed between a linear polarizer at an angle α and a circular analyser is given by 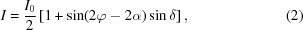
where *I*_0_ is the intensity of unpolarized light passing through the sample, α is the angle of the linear polarizer, and φ is the angle measured anticlockwise from the horizontal direction of the microscope stage to the slow axis of the indicatrix of the material under investigation. δ is the phase difference experienced by perpendicular optical polarizations through a material of thickness *t* and is given by 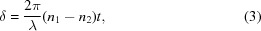
where λ is the wavelength of the incident light and *n*_1_ and *n*_2_ are the refractive indices of the light while propagating through the material. To measure the intensity of the transmitted light, a CCD detector is used containing 1360 × 1024 pixels. Each pixel forms a separate linear polarizer and analyser system; thus the CCD provides 1.4 million separate measurements of the transmitted light intensity. By recording *I*_0_ for a range of linear polarizer angles, α, equation (2) can be solved for *I*_0_, φ and |sin δ|. False colours are then applied to each of these quantities to produce separate maps of *I*_0_, φ and |sin δ|.

**Figure 1:**
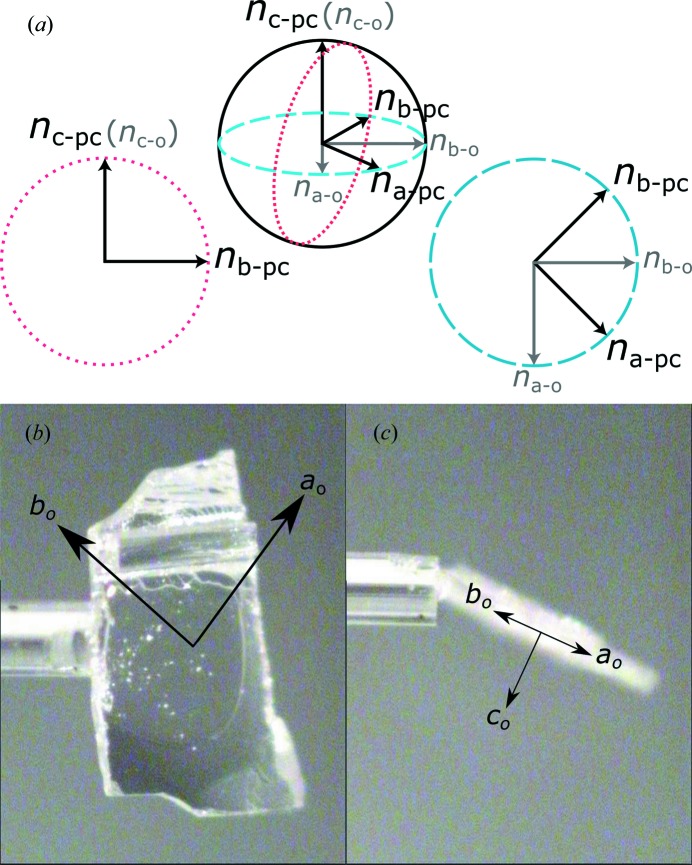
(*a*) The optical indicatrix of an orthorhombic system in the rhombic orientation, viewed down [100]_pc_ and [001]_pc_, is shown in dotted red and dashed blue lines, respectively. Orthorhombic refractive indices are shown in grey and pseudocubic in black. The orientation of the orthorhombic axes in one of the crystal plates used for the Metripol measurements is shown in (*b*) and (*c*).

The optical indicatrix of an orthorhombic crystal (drawn with a small difference between the refractive indices) is shown in Fig. 1 ▸(*a*). Sections of the indicatrix when viewed down [100]_pc_ and [001]_pc_ are shown as dotted red and dashed blue lines, respectively. When viewed down [001]_pc_ the birefringence is given by the difference between the orthorhombic *n*_*a*-o_ and *n*_*b*-o_, and down [100]_pc_ by the difference between *n*_*c*-o_ and *n*_*b*-pc_ {or 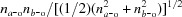 calculated using the equation of an ellipse}.

Two crystal plates of PbHfO_3_ were used in this study, one for high-resolution X-ray diffraction and the other for the Metripol measurements. The orientation of the crystal was determined using an Oxford Diffraction Gemini diffractometer equipped with a Mo *K*α source. BIM measurements were made using the Metripol system at 1 K intervals from 300 to 493 K, during both heating and cooling. Repeated heating and cooling cycles were performed on the same crystal without changing the orientation of the crystal with respect to the microscope between runs.

High-resolution X-ray diffraction measurements were performed on a PANalytical X’Pert Pro MRD equipped with a hybrid mirror–monochromator, providing an intense source of Cu *K*α_1_ radiation. An Anton Paar DHS 1100 furnace was used for measurements at high temperatures. A PIXcel detector in conjunction with a Ge analyser crystal was used to enable precise measurements in 2θ of the positions of the measured Bragg peaks. The lattice parameters were calculated in the room-temperature orthorhombic phase by measurements of the 400_o_, 080_o_ and 244_o_ reflections. The 400­_o_, 322_o_ and 244_o_ reflections were measured in the intermediate phase, and 244_o_ (202_pc_) in the cubic phase.

The crystal plate for X-ray diffraction measurements was attached to a piece of an Si wafer using silver paste and then mounted into the furnace. A 2 mm beam mask and 1/16th degree slit were used to ensure that the incident X-ray beam was of a similar size to the crystal. This allowed the crystal to be accurately placed in the centre of the beam and at the centre of rotation of the diffractometer. At each measurement temperature the height of the crystal was corrected to ensure it remained at the centre of the beam.

## 3. Results and discussion   

### 3.1. Orientation   

Before carrying out the Metripol measurements one of the crystal plates was mounted onto the end of a glass fibre using Araldite and mounted in the Oxford Diffraction Gemini diffractometer. A number of reflections were measured at room temperature and analysed using the *CrysAlis* software. The unit cell suggested by the software was orthorhombic with *a* = 5.84 (1), *b* = 11.70 (1), *c* = 8.19 (1) Å, α = 90.0 (1), β = 89.9 (1) and γ = 89.9 (1)°, which agrees well with the values of *a* = 5.8404 (3), *b* = 11.7057 (5) and *c* = 8.1751 (1) Å determined by Madigout *et al.* (1999 ▸) using powder neutron diffraction. The relationship between the orthorhombic lattice parameters and the crystal plate is shown in Figs. 1 ▸(*b*) and 1 ▸(*c*).

### 3.2. BIM measurements   

**Figure 2:**
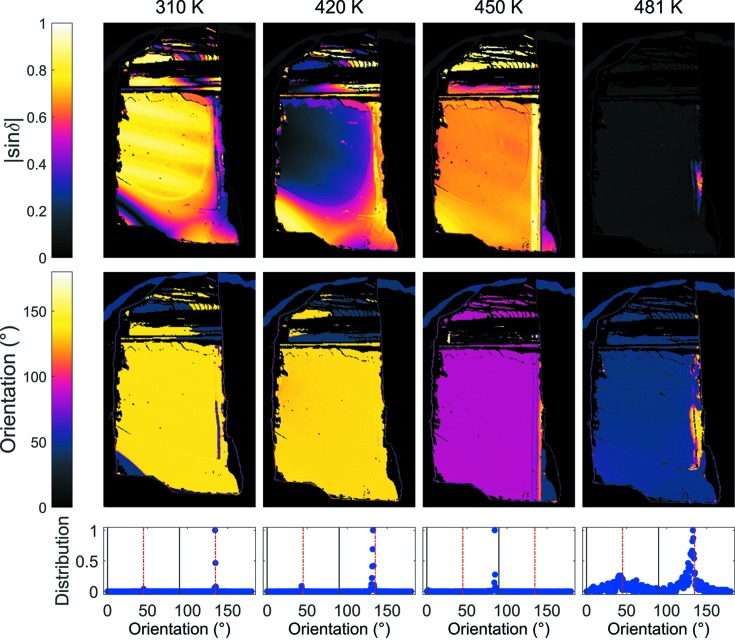
Measured images of the |sin δ|, orientation and orientation distributions at 310, 420, 450 and 481 K during heating. Orientations consistent with tetragonal and rhombohedral symmetries are marked by the black and dashed red lines, respectively. Orthorhombic orientations are consistent with both. A red–green colour perception deficient colour scheme taken from Geissbuehler & Lasser (2013 ▸) is used.

The crystal was placed in the microscope while attempting to align a pseudocubic direction with the horizontal plane of the microscope; this was however difficult to do precisely since the crystal did not have any straight edges with a similar orientation. This means that the determined orientations are likely to vary by ±5° from the expected values, and movement as a function of temperature could also result in some deviation. A video showing |sin δ| and orientation images as a function of temperature can be downloaded from the supporting information for this paper. Images of the measured |sin δ|, the orientation and the distribution of the orientation angles at 310, 420, 450 and 481 K are shown in Fig. 2 ▸. The |sin δ| images are coloured black for low birefringence and also artificially for pixels not on the sample and areas that did not have sufficient light transmission for analysis. The yellow stripes in the |sin δ| image at 310 K are an interference effect usually introduced when the bottom of the sample is not perfectly parallel with the glass plate on which it is placed.

In the *A*_1_ phase the orientation was 134 (2)° from one of the pseudocubic axis directions, which has been aligned with the horizontal axis of the Metripol. Note that the figure in parentheses represents the error in measuring the orientation from the images, but the actual value may be incorrect by ±5° as mentioned above. This angle is close to 135° and is consistent with either rhombohedral or orthorhombic symmetries. At 450 K in the *A*_2_ phase, the main area of the crystal has an orientation of 85 (2)°, *i.e.* close to 90°, along with a small domain in the bottom right with an orientation of 45 (2)°. Having domains with orientations close to 45 and 90° points to the crystal structure of this phase having a unit cell that is orthorhombic in the rhombic orientation. At 481 (1) K the crystal is cubic and the measured |sin δ| is of the order of the background value for the microscope except for a small area which still has a measurable birefringence. This probably arises from an increased thickness in this section resulting in a cooler area on the surface furthest from the heating element of the furnace.

**Figure 3:**
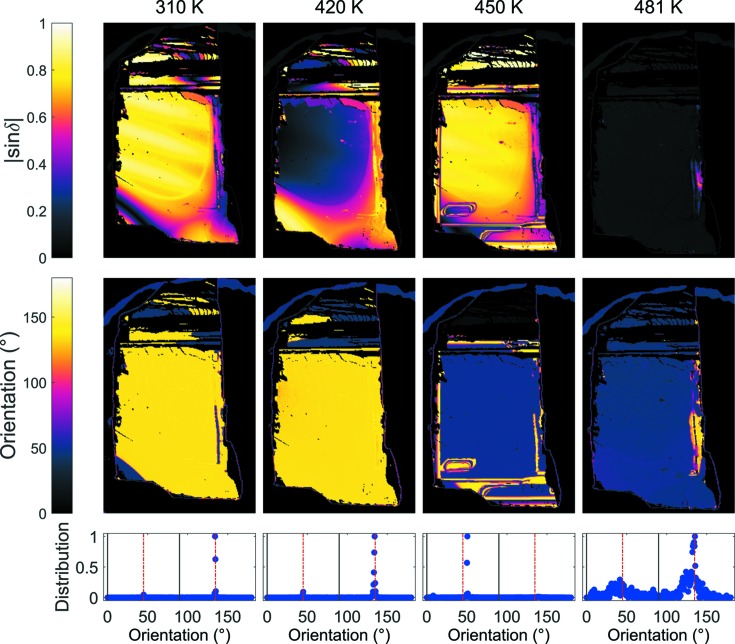
Measured images of the |sin δ|, orientation and orientation distributions at 310, 420, 450 and 481 K during cooling. Orientations consistent with tetragonal and rhombohedral symmetries are marked by the black and dashed red lines, respectively. Orthorhombic orientations are consistent with both.

The BIM measurements were repeated during cooling of the crystal and are shown in Fig. 3 ▸. On cooling the orientation in the *A*_2_ phase is predominantly at 51 (2)°, with a small section at the top of the crystal at 8 (2)°. These are close to 45 and 0°, again confirming that the symmetry is orthorhombic in the rhombic orientation, and neither rhombohedral nor tetragonal.

**Figure 4:**
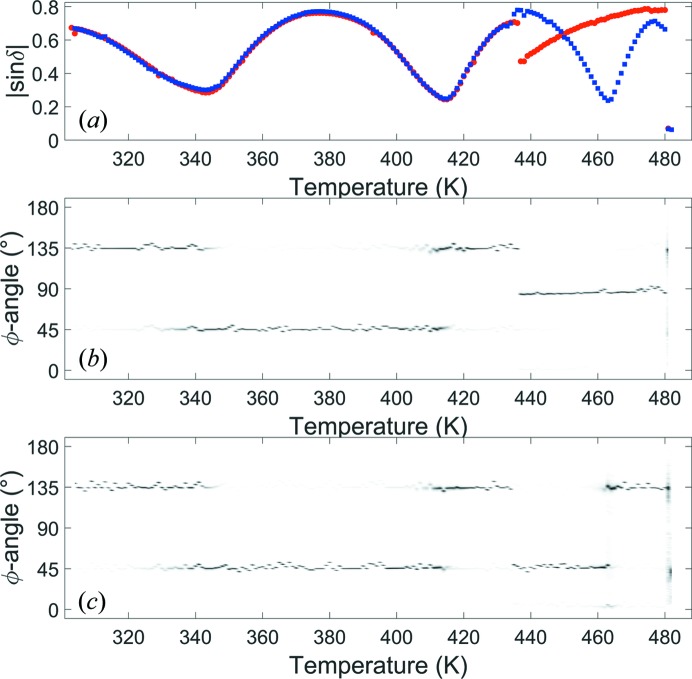
(*a*) The average |sin δ| given by red circles and blue squares during heating and cooling, respectively, and measured distributions of φ during (*b*) heating and (*c*) cooling.

The average |sin δ| of the crystal is plotted in Fig. 4 ▸(*a*), with the data coloured red and blue on heating and cooling, respectively. The distribution of the orientation angle across the whole crystal is plotted during heating in Fig. 4 ▸(*b*) and cooling in Fig. 4 ▸(*c*). The measured values of |sin δ| in the *A*_1_ phase, below 430 K, are the same on heating and cooling. In this phase the orientation angle varies by 90° at ∼340 K and ∼415 K where the |sin δ| signal goes through a minimum. This occurs as the birefringence increases or decreases resulting in the value of δ being equal to ±*m*π, where *m* is an integer.

The phase change to the *A*_2_ phase shows the expected hysteresis with the transition occurring at 437 (1) K on heating and 434 (1) K on cooling. On heating, an abrupt drop in the measured |sin δ| signal and a change in orientation of ∼45° are observed. The transition on cooling does not show a significant change in |sin δ| and the orientation change is now 90°. This gives an orientation difference of ∼45° between the domains in the *A*_2_ phase on heating and cooling as shown in Figs. 4 ▸(*b*) and 4 ▸(*c*).

In the *A*_2_ phase the domains formed during heating and cooling also have a different |sin δ| response as a function of temperature. The rate of change during cooling is much greater than on heating, showing that the measured birefringence is not from the same crystallographic directions during heating and cooling. The transition to the cubic phase from the *A*_2_ phase occurs at 481 (1) K, with a low |sin δ| signal measured across the crystal. On cooling, the transition to the *A*_2_ phase occurs at 480 (1) K, showing a small hysteresis consistent with the literature.

### 3.3. High-resolution X-ray diffraction   

**Figure 5:**
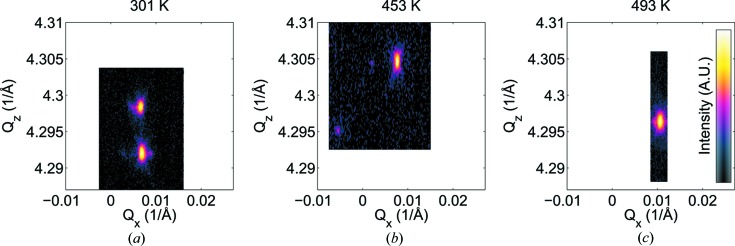
Reciprocal-space map of (*a*) the 080_o_ and 400_o_ reflections at 301 K, (*b*) 080_o_/400_o_ and 244_o_ at 453 K, and (*c*) 244_o_ at 493 K.

In the room-temperature orthorhombic phase, the measured out-of-plane reflection corresponded to 240_o_, confirming that the *c*_o_ axis is in-plane. The lattice parameters in this phase were calculated using the 080_o_, 400_o_ and 244_o_ reflections. The reciprocal-space map measured at 301 K at the expected position of the 080_o_ reflection is shown in Fig. 5 ▸. This consists of two peaks, with one from the expected 080_o_ reflection and the other from 400_o_, showing that this phase forms with 90° domains.

In the *A*_2_ phase, the 080_o_ and 400_o_ peaks are replaced by a single weak peak and the appearance of a {244_o_}-type reflection. This becomes visible because of a change in the domain structure to a domain with a *c*_o_ component out-of-plane. This was confirmed by the presence of the 004_o_ reflection along with 240_o_ in the out-of-plane reflections.

**Figure 6:**
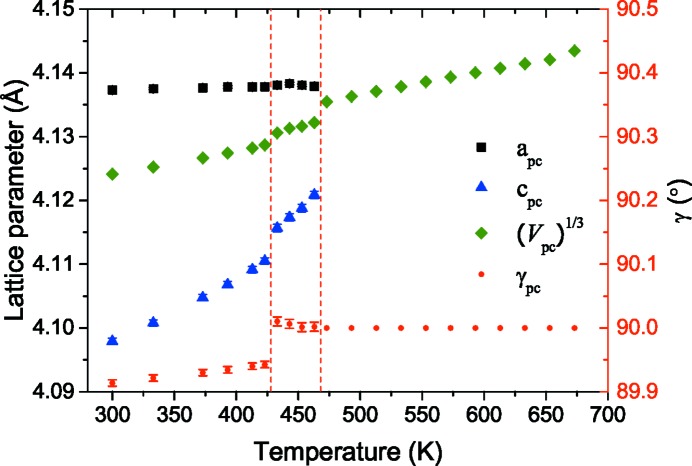
The pseudocubic lattice parameters as a function of increasing temperature. *a*_pc_, *c*_pc_, (*V*_pc_)^1/3^ and γ_pc_ are marked by black squares, blue triangles, green diamonds and red circles, respectively. Error bars are calculated from the uncertainty of the reflection positions in 2θ and are smaller than the symbols for the lattice parameters.

The pseudocubic lattice parameters are plotted along with the γ_pc_ angle as a function of temperature in Fig. 6 ▸. The errors on the measured lattice parameters are smaller than the size of the data points and were calculated from the uncertainties in the positions of the measured Bragg reflections. *a*_pc_ = *b*_pc_ remains relatively constant as a function of temperature until the transformation to the cubic phase, when the lattice parameter drops. As the temperature increases towards the intermediate phase, γ_pc_ increases linearly from 89.91 (1)° to 89.95 (1)° and jumps very close to 90° in the *A*_2_ phase.

*c*_pc_ increases linearly in both the initial orthorhombic phase and the intermediate phase with a step between them. The rate of change in the intermediate phase is greater than that seen in the orthorhombic phase. The larger variation in *c*_pc_ compared with *a*_pc_ = *b*_pc_ is consistent with the BIM results in the *A*_2_ phase, which showed a greater change in birefringence as a function of temperature when the *c*_o_ direction was in-plane during cooling in Fig. 4 ▸.

In the *A*_2_ phase the measured birefringence is given by the difference between *n*_*a*-o_ and *n*_*b*-o_ and *n*_*c*-o_ and 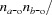 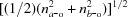, during heating and cooling, respectively. The change in |sin δ| and orientation as a function of temperature shows that the rate of change of the birefringence is greater between *n*_*c*-o_ and 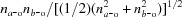 than between *n*_*a*-o_ and *n*_*b*-o_ in this phase.

These results were repeatable on subsequent heating and cooling cycles; the domain structure in the room-temperature and intermediate phases varied between each cycle but the phase transition temperatures were consistent. The phase transition between the room-temperature orthorhombic phase and the *A*_2_ phase showed a thermal hysteresis with the phase transition occurring at 437 (1) and 434 (1) K on heating and cooling, respectively. A small thermal hysteresis was measured in the transition between the *A*_2_ and the cubic phase. These values are consistent with previous measurements made investigating the phase transitions of PbHfO_3_.

## 4. Conclusions   

In conclusion, we have carried out a detailed study of the phase transitions of PbHfO_3_ with a focus on determining the symmetry of the intermediate *A*_2_ phase. High-resolution X-ray diffraction measurements limit the possible space groups of the phase to either orthorhombic or tetragonal crystal systems, whereas the domain orientations measured using BIM are only consistent with orthorhombic symmetry in the rhombic orientation. The phase transition temperatures and thermal hysteresis measured using BIM and X-ray diffraction are in agreement with the previous values determined in the literature. The combination of X-ray diffraction and BIM provides an unambiguous method for determining the symmetry of crystals.

It is worth noting that the anomaly observed in the temperature dependence of the permittivity close to the transition from the *A*_2_ to the paraelectric phase, as reported by Bussmann-Holder *et al.* (2015 ▸), is not observed in these optical measurements. This is consistent with their assertion that this anomaly is induced by the electric field used in measurements of permittivity and most probably connected with a movement of domain boundaries in that electric field. At higher intensities of electric field, a saturated hysteresis loop was observed in the same temperature region as that in which this anomaly develops, additional proof of the induced character of the phase that may appear just below *T*_c_ in dielectric measurements.

## Supplementary Material

Video of |sindelta| and orientation images as a function of temperature.. DOI: 10.1107/S1600576717000309/aj5287sup1.avi

## Footnotes

1 The subscript pc refers to the use of pseudocubic perovskite axes.
